# Diagnostic Test Accuracy of Artificial Intelligence in Detecting Periapical Periodontitis on Two-Dimensional Radiographs: A Retrospective Study and Literature Review

**DOI:** 10.3390/medicina59040768

**Published:** 2023-04-15

**Authors:** Julien Issa, Mouna Jaber, Ismail Rifai, Paul Mozdziak, Bartosz Kempisty, Marta Dyszkiewicz-Konwińska

**Affiliations:** 1 Department of Diagnostics, University of Medical Sciences, Bukowska 70, 60-812 Poznan, Poland; m.dyszkiewicz@ump.edu.pl; 2 Doctoral School, Poznań University of Medical Sciences, Bukowska 70, 60-812 Poznan, Poland; 3 Faculty of Dentistry, Poznan University of Medical Sciences, 60-812 Poznan, Poland; 82671@student.ump.edu.pl; 4 Department of Restorative Dentistry and Endodontics, Universitat Internacional de Catalunya, Josep Trueta, s/n, 08195 Sant Cugat del Vallès, Spain; ismailrifai@uic.es; 5 Prestage Department of Poultry Sciences, North Carolina State University, Raleigh, NC 27695, USA; pemozdzi@ncsu.edu; 6 Physiology Graduate Faculty, North Carolina State University, Raleigh, NC 27695, USA; kempistybartosz@gmail.com; 7 Division of Anatomy, Department of Human Morphology and Embryology, Wroclaw Medical University, Chalubinskiego 6a, 50-368 Wroclaw, Poland; 8 Department of Veterinary Surgery, Institute of Veterinary Medicine, Nicolaus Copernicus University in Torun, Gagarina 7, 87-100 Torun, Poland; 9 Center of Assisted Reproduction, Department of Obstetrics and Gynaecology, University Hospital and Masaryk University, Jihlavska 20, 62500 Brno, Czech Republic

**Keywords:** artificial intelligence (AI), periapical periodontitis, diagnosis, two-dimensional radiographs, diagnostic test accuracy, automatic detection

## Abstract

This study aims to evaluate the diagnostic accuracy of artificial intelligence in detecting apical pathosis on periapical radiographs. A total of twenty anonymized periapical radiographs were retrieved from the database of Poznan University of Medical Sciences. These radiographs displayed a sequence of 60 visible teeth. The evaluation of the radiographs was conducted using two methods (manual and automatic), and the results obtained from each technique were afterward compared. For the ground-truth method, one oral and maxillofacial radiology expert with more than ten years of experience and one trainee in oral and maxillofacial radiology evaluated the radiographs by classifying teeth as healthy and unhealthy. A tooth was considered unhealthy when periapical periodontitis related to this tooth had been detected on the radiograph. At the same time, a tooth was classified as healthy when no periapical radiolucency was detected on the periapical radiographs. Then, the same radiographs were evaluated by artificial intelligence, Diagnocat (Diagnocat Ltd., San Francisco, CA, USA). Diagnocat (Diagnocat Ltd., San Francisco, CA, USA) correctly identified periapical lesions on periapical radiographs with a sensitivity of 92.30% and identified healthy teeth with a specificity of 97.87%. The recorded accuracy and F1 score were 96.66% and 0.92, respectively. The artificial intelligence algorithm misdiagnosed one unhealthy tooth (false negative) and over-diagnosed one healthy tooth (false positive) compared to the ground-truth results. Diagnocat (Diagnocat Ltd., San Francisco, CA, USA) showed an optimum accuracy for detecting periapical periodontitis on periapical radiographs. However, more research is needed to assess the diagnostic accuracy of artificial intelligence-based algorithms in dentistry.

## 1. Introduction

Periapical lesions are bony lesions located around the tooth root apex [1] whose origin can be inflammatory (periapical granuloma and radicular cyst) or non-inflammatory (cemental periapical dysplasia, keratocyst, etc.) [2]. The inflammatory periapical lesions are the most common, mainly induced by bacterial infection, most predominantly by Gram-negative bacteria [3]. These lesions are frequently the result of untreated dental decay, tooth wear, and dental trauma leading to pulp necrosis [4]. Periapical granulomas and radicular cysts are associated with apical periodontitis (apical lesions) and can be histologically differentiated by the absence or presence of an epithelial lining, respectively [5]. Based on a meta-analysis by Tibúrcio-Machado et al. [6], the global frequency of teeth with apical periodontitis is 5%, with the highest prevalence in individuals from transitional and developing countries. Apical periodontitis can either be asymptomatic and therefore identified accidentally during a routine dental radiological evaluation or symptomatic, causing symptoms such as discoloration, sensitivity, pain, swelling, and drainage [4,7]. In severe cases, the infection can spread into the surrounding tissues, causing serious health problems such as space infection [4,8]. Space infection occurs when an infection spreads to the deep spaces within the head and neck and potentially results in life-threatening conditions (airway compromise, pericarditis, etc.) [8]. 

The manifestation of apical periodontitis may include loss of the periodontal ligament continuity, periodontal ligament space widening, and resorption of periapical bone, leading to periapical radiolucency on dental X-rays [9,10,11]. A periapical radiograph (periapical X-ray) using the paralleling technique is the projection of choice for diagnosing apical lesions, providing a complete image of the tooth and its surroundings as the periapical region, periodontium, etc. [12,13,14]. The paralleling technique requires aligning the X-ray film or sensor with the long axis of the tooth, and the X-ray beam is directed perpendicular to the X-ray film or sensor [15]. This technique produces an image with less superimposition and distortion [16]. Nevertheless, periapical X-ray interpretation can sometimes be challenging, as the lesions cannot be easily detectable on the X-ray due to changes in bone density (thinner bone) [12], complicated anatomical location [17], or insufficient dentist experience.

Artificial intelligence (AI) describes the ability of machines to perform functions that are typically accomplished by humans [18]. Machine learning (ML) is a subgroup of AI that enables computers to learn from data and make predictions based on this process that can analyze large datasets, allowing the algorithm to learn from the interpreted data and improve its performance over time [19]. Consecutively, deep learning (DL) is a form of ML that uses artificial neural networks (ANNs) that work in the same way as the human brain [20]. They are designed to process large amounts of data and learn from it [20]. Convolutional neural networks (CNNs) are a type of ANN that is particularly well-suited for image classification tasks [21,22]. They are designed to analyze the spatial relationships between elements in an image and extract useful information [21,22]. CNNs have been used in many applications, such as recognizing objects in images, classifying images into different categories, and extracting meaningful information from images [21,22]. Recently, there has been a growing interest in utilizing artificial intelligence, particularly deep learning (DL) and machine learning (ML), for diagnosing medical and dental conditions. These technologies have the potential to enhance the accuracy of diagnoses, reduce the time required for diagnosis, and ultimately lead to cost savings. AI has been tested for various applications in dentistry, showing promising results, including for detecting dental caries [23], screening for oral cancer [24], tooth numbering [25], and segmenting the inferior alveolar nerve [26], among others.

Additionally, the recent approval of several AI tools for dental image analysis by the Food and Drug Administration (FDA) [27] is a significant step forward in the clinical use of AI in dentistry. However, despite these advancements, it is important to note that the clinical use of AI in dentistry is still in its early stages, and more research is needed to establish its effectiveness and accuracy. In particular, there is a need for further evidence to support the use of AI in periapical lesion diagnosis on periapical X-rays. In this study, we aimed to evaluate the diagnostic accuracy of artificial intelligence in detecting apical pathosis on periapical radiographs.

## 2. Literature Review 

After conducting a comprehensive literature search and analysis (Table 1), we found seven studies that trained and tested AI-based tools for detecting apical lesions on periapical X-rays [28,29,30,31,32,33,34]. 

Of these seven studies, two [30,32] reported the sensitivity, specificity, and accuracy of the algorithms, while two others [28,29] reported only the sensitivity and specificity of the algorithms. The F1 score, which measures the balance between precision (positive predictive value) and recall (sensitivity), was only reported by two studies [29,30]. 

Hamdan et al. [28] used 68 periapical radiographs in their study to test a CNN model (Denti.AI) that achieved a sensitivity of 93.1% by case and 88.8% by lesion, respectively. This model also showed a specificity of 73.3% (by case) [28]. In another retrospective study, Li et al. [29] used a total of 4129 periapical radiographs to train and test the AI model constructed based on ResNet-18. The model showed 82% sensitivity, 84% specificity, and an F1 score equal to 0.82 [29]. The sensitivity, specificity, accuracy, and F1 score recorded with YOLO (You Only Look Once) version 3 in the study by Moidu et al. [30] performed on 1950 periapical radiographs were 92.1%, 76%, 86.3%, and 0.89, respectively. Li et al. [32] used 476 periapical X-ray images to train, validate, and test a CNN-based model that achieved a sensitivity of 94.87%, specificity of 90%, and 92.75% accuracy. 

An analysis of the demographic distribution of the retrieved studies showed that China was the largest contributor, with two studies [29,31], while the other studies came from the United States [28], Taiwan [32], India [30], Canada [33], and Italy [34].

## 3. Materials and Methods

### 3.1. Image Data Set

Twenty anonymized digital periapical radiographs were carefully selected in this retrospective study based on specific inclusion criteria. These radiographs were collected from 20 patients (10 male and 10 female) and were taken at the Department of Diagnostics at Poznan University of Medical Sciences between March 2022 and May 2022. 

The radiographs were acquired with a Digora Optime (Soredex/Orion Corp., Helsinki, Finland) storage phosphor plate (SPP) system with an exposure time of 0.125 s, an X-ray tube voltage of 70 kV, and an X-ray tube current of 7 mA. The digital radiographs were afterward scanned with the Digora Optime system. The obtained X-ray images were processed using the software provided by the system with no filters added to the images during the processing stage to obtain unaltered radiographs.

To be eligible for inclusion, the scans had to meet the following criteria: taken of patients over 18 years of age, scan of permanent teeth, having high-resolution quality, and clearly showing at least one tooth with the periodontium. Conversely, scans with any type of artifact, such as a cone-cut or motion artifact, as well as radiographs of deciduous teeth, those taken from patients under 18 years of age, and those of poor quality where the periodontium of the teeth was not visible were excluded from the study.

### 3.2. Ground-Truth Evaluation 

**Figure 1:**
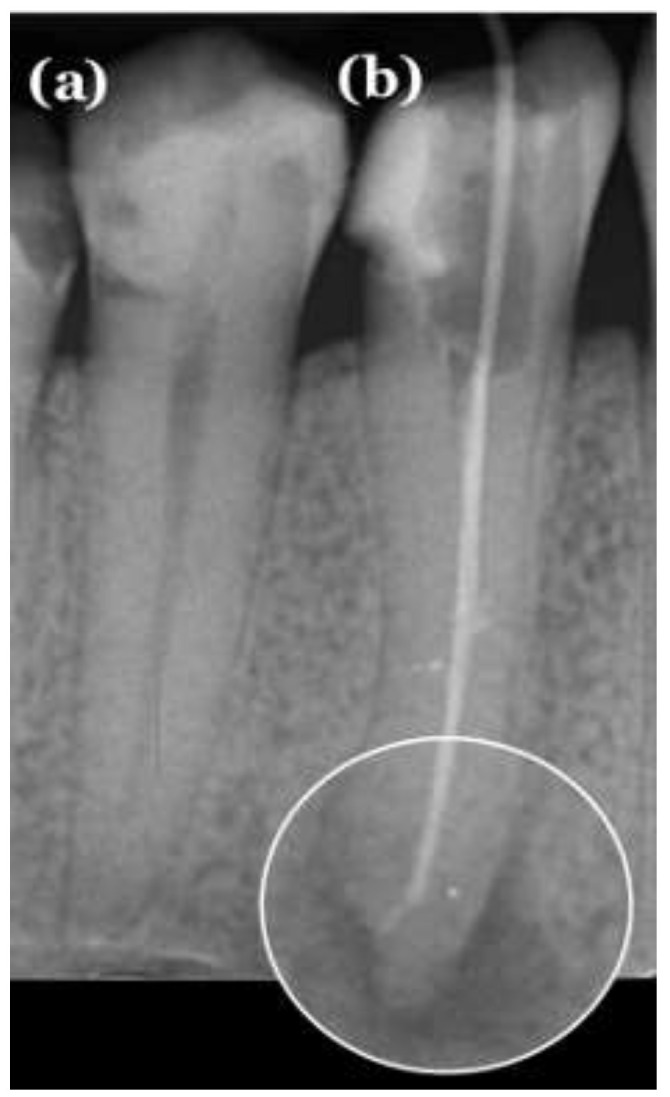
Periapical radiograph showing two mandibular premolars with the periodontium. The investigators classified the teeth as (**a**) healthy and (**b**) unhealthy.

Two investigators, including an oral and maxillofacial radiology expert with more than ten years of experience (M.D.K) and one trainee in oral and maxillofacial radiology (J.I), performed an independent manual radiographic evaluation that was considered as the ground-truth evaluation. The evaluation process involved identifying the presence or absence of periapical radiolucency based on the periapical index (PAI) [35], which includes five scores. The radiographs were analyzed using a certified medical monitor with a proper resolution to enable detailed examination. A tooth was considered healthy when no periapical radiolucency was detected, scoring one on the PAI (Figure 1). If a periapical radiolucency was present, the tooth was classified as unhealthy, scoring from two to five on the PAI (Figure 1). To ensure the validity of the results, the evaluation process was recorded separately by each investigator and repeated twice with a seven-day interval between repetitions. Subsequently, the inter-rater and intra-rater reliability was calculated to determine the level of consistency between the evaluations performed by the two investigators and the consistency within the evaluations conducted by each investigator, respectively. 

### 3.3. Automated Evaluation

**Figure 2:**
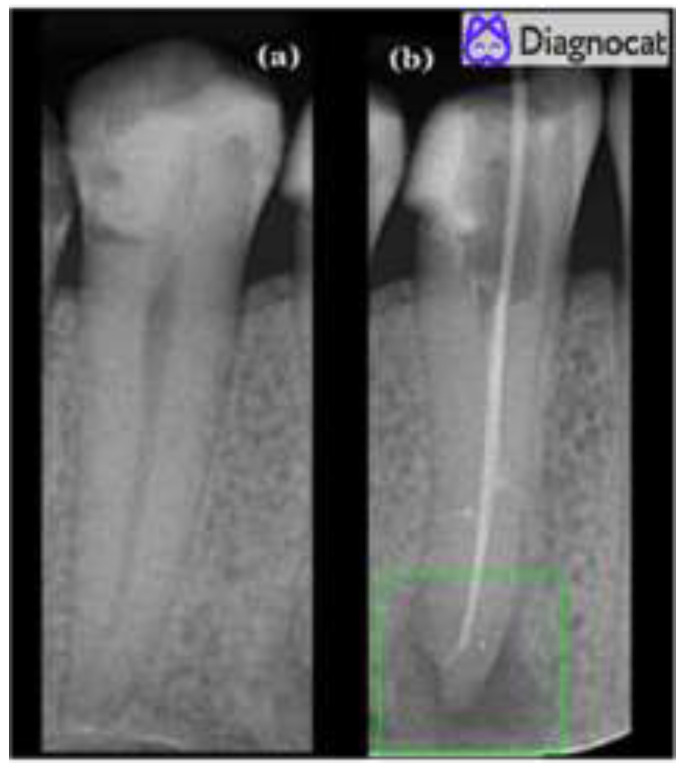
Periapical radiograph showing two mandibular premolars with the periodontium. Diagnocat (Diagnocat Ltd., San Francisco, CA, USA) classified the teeth as (**a**) healthy and (**b**) unhealth, marked by a green square.

The same periapical radiographs were uploaded to Diagnocat (Diagnocat Ltd., San Francisco, CA, USA), a cloud-based AI tool. This tool is designed to store and process dental images using a U-net-like architecture. For each radiograph, a report was generated by Diagnocat (Diagnocat Ltd., San Francisco, CA, USA), which highlighted the periapical lesions on the unhealthy teeth (Figure 2). On the other hand, no highlights were observed on healthy teeth (Figure 2). The results were recorded and then compared to the ground-truth method evaluation results to determine the accuracy of Diagnocat (Diagnocat Ltd., San Francisco, CA, USA).

## 4. Results

The inter-rater reliability of the analysis was found to be 98%, indicating a remarkable level of agreement between the two investigators. The examination of the 60 teeth by both investigators almost reached a consensus on the diagnoses, with only a minimal difference in their assessments. Moreover, an absolute agreement in the repeated evaluation was recorded for each rater (intra-rater reliability = 100%). 

The assessment of the 60 teeth by the experts led to the classification of 13 teeth as unhealthy and 47 as healthy teeth. This was in line with the results produced by the AI-based tool, Diagnocat (Diagnocat Ltd., San Francisco, CA, USA, which also identified 13 teeth as unhealthy and 47 as healthy. 

In order to evaluate the accuracy of the AI-based tool, the produced results were compared to the results of the ground-truth method. The results of this comparison are displayed in Table 2 and are represented as diagnostic odds:

- Twelve true positives (TP) (teeth with apical periodontitis, unhealthy);
- One false positive (FP) (tooth with no signs of apical periodontitis was classified as unhealthy, over-diagnosed by the tool);
- Forty-six true negatives (TN) (healthy teeth, no signs of any apical periodontitis);
- One false negative (FN) (misdiagnosed by the tool; a tooth with apical periodontitis was classified as a healthy tooth).

Furthermore, the diagnostic odds ratios (sensitivity, specificity, and accuracy) and F1 score were calculated based on the following equations:

- Sensitivity = TP/FP + TP
- Specificity = TN/FN + TN
- Accuracy = TP + TN/TP + TN + FP + TP
- F1 score = 2 ∗ TP / (2 ∗ TP + FP + FN)

Diagnocat (Diagnocat Ltd., San Francisco, CA, USA) scored a sensitivity of 92.30%, 97.87% specificity, 96.66% accuracy, and 0.92 F1 score.

## 5. Discussion

The application of AI in dentistry has been on the rise in recent years, with its application being observed in various dental specialties. Despite this trend, the number of studies investigating the diagnostic accuracy of AI-based tools in various conditions and locations remains relatively limited. 

However, given the potential benefits that AI-based tools can offer in terms of accuracy, speed, and sustainability, it is important that research in this area continues to grow and expand. Such studies will provide a better understanding of the capabilities and limitations of these tools and help to guide their development and application in the future. 

In the field of endodontics, the potential of AI to detect periapical lesions has been tested through various imaging techniques. This includes cone-beam computed tomography (CBCT) scans [36,37,38,39], panoramic images [40,41,42,43,44], as well as periapical X-rays (Table 1). The current standards for diagnosing periapical lesions involve a clinical evaluation alongside taking a periapical radiograph, which dentists and specialists routinely perform. Considering the clinical importance of periapical radiographs and the potential clinical use of AI for detecting apical periodontitis, we conducted a retrospective study to assess the diagnostic performance of AI in detecting periapical pathosis on periapical radiographs. To the best of our knowledge, our study is the first to evaluate the diagnostic performance of Diagnocat (Diagnocat Ltd., San Francisco, CA, USA) in detecting apical periodontitis on periapical X-rays using this specific methodology.

In our study, the U-net-like based tool that we tested, Diagnocat (Diagnocat Ltd., San Francisco, CA, USA), demonstrated significant accuracy in identifying apical periodontitis and healthy teeth. The tool correctly identified 12 (true positive) cases of apical periodontitis, which resulted in a high sensitivity of 92.30%. This high sensitivity demonstrates that Diagnocat could accurately identify the presence of periapical lesions in most cases. Additionally, Diagnocat (Diagnocat Ltd., San Francisco, CA, USA) was able to identify 46 (true negative) healthy teeth, leading to a significant specificity of 97.87%. This high specificity indicates that the tool could accurately identify the absence of periapical lesions in most cases. Another important metric used to evaluate the performance of Diagnocat (Diagnocat Ltd., San Francisco, CA, USA) was the F1 score. The F1 score measured the accuracy of the tool in identifying periapical lesions while balancing the number of false negatives and false positives. In our study, Diagnocat (Diagnocat Ltd., San Francisco, CA, USA) achieved a high F1 score of 0.92, indicating that the tool could accurately identify periapical lesions with a low rate of false negatives and false positives. Finally, Diagnocat (Diagnocat Ltd., San Francisco, CA, USA) was able to detect the presence or absence of apical periodontitis with high accuracy, with a recorded accuracy of 96.66%.

On the other hand, a study conducted by Zadrozny et al. [44] tested the diagnostic accuracy of Diagnocat in detecting periapical lesions on 30 panoramic radiographs, which included a total of 805 teeth. The sensitivity of Diagnocat in this study was recorded at 30.9%, which is considered to be low. However, the specificity of Diagnocat was found to be very high at 98.1%. In terms of diagnosing periapical lesions, the results of the study showed that Diagnocat was able to correctly diagnose (true positive) 23 lesions, but it also misdiagnosed (false negative) 36 lesions and over-diagnosed (false positive) 14 lesions. The results obtained by this study [44] were different from the results obtained in our study. This difference could be due to various factors, such as poor training of the algorithm on detecting periapical lesions on panoramic images, anatomical considerations, and differences in radiographic accuracy, as the periapical radiographs offer a smaller field of view with better resolution [45,46]. Orhan et al. [39] conducted a study that evaluated the performance of the Diagnocat (Diagnocat Ltd., San Francisco, CA, USA) algorithm in identifying periapical lesions on CBCT scans. The sample for this study consisted of 153 CBCT scans from 109 different patients, all of whom had periapical lesions. The results showed that the Diagnocat algorithm was able to accurately identify 142 of these lesions, resulting in a reliability of 92.8%. In terms of the algorithm’s recall and precision, the estimated recall was 89%, while the estimated precision was 95%. This high level of precision and recall demonstrates the effectiveness of the Diagnocat algorithm in accurately identifying periapical lesions on CBCT scans. However, it should be noted that the sample in this study was limited to CBCT scans with periapical lesions.

This study has a number of limitations that should be taken into consideration when interpreting the results. Firstly, the sample size used in this study is small, with 20 periapical radiographs and 60 teeth being evaluated. 

Another limitation of this study is the unequal distribution of healthy and unhealthy teeth. This imbalance in the sample could impact the sensitivity and specificity values, which are important indicators of the accuracy of the diagnostic test being used.

Additionally, the data used in this study were collected from a single database (Poznan University of Medical Sciences) and were all registered using the same device. While the results obtained in this study are interesting, it is possible that different results may be obtained if data from different locations were used and if other devices were used for the registration. To further validate the findings, collecting data from multiple sources and testing the results using different devices would be important.

While this study provides valuable insights into the investigated area, the abovementioned limitations must be considered when interpreting the results. Further research using larger sample sizes and data from different sources would help to strengthen the validity and reliability of the findings.

## 6. Conclusions

The evaluated AI algorithm demonstrated an optimum accuracy for detecting apical periodontitis on periapical radiographs, as the algorithms can discover patterns and characteristics that the human eye cannot. It allows it to be used as a fast-assisting diagnostic tool for dentists while lowering clinical effort and improving treatment quality. The next stage is to test the algorithm’s effectiveness in following up on the healing progress of the detected lesion after treatment. 

The application of AI in detecting periapical lesions in endodontics is a rapidly growing area of research. More studies are needed to further explore the capabilities and limitations of these tools to understand better the role of AI in improving the accuracy and efficiency of the diagnostic process and ultimately improving patient outcomes.

## Figures and Tables

**Table 1:** Studies testing the application of AI in detecting apical lesions on periapical radiographs.

Author, Study Location, and Year of Publication	Sample Size (Periapical Radiographs)	X-ray Technology	AI Model	Sensitivity	Specificity	Accuracy	F1 Score
Hamdan et al., United States, 2022 [28]	68	Photostimulable phosphor (PSP) plates scanned using ScanX (Air Techniques, Hicksville, NY, USA), Soredex Digora Optime (Kavo Dental, Charlotte, NC, USA) Sirona Schick33 Direct Digital Sensor (Dentsply Sirona, Charlotte, NC, USA) XDR Anatomic Sensor (Cyber Medical Imaging, Los Angeles, CA, USA).	CNN (Denti.AI)	93.1% (by case) 88.8% (by lesion)	73.3% (by case)	N/A	N/A
Li et al., China, 2022 [29]	4129	N/A	ResNet-18	82%	84%	N/A	0.82
Moidu et al., India, 2022 [30]	1950	Size-2 CMOS RVG sensor (Kodak RVG 5100, Eastman Kodak Company, France)	YOLO (You Only Look Once) version 3	92.1%	76%	86.3%	0.89
Chen et al., China, 2021 [31]	2900	Digital	Fast-R-CNN (Fast Region-based convolutional neural network)	N/A	N/A	N/A	N/A
Li et al., Taiwan, 2021 [32]	476	N/A	CNN	94.87%	90%	92.75%	N/A
Li et al., Canada, 2007 [33]	60	N/A	SVM (support vector machine)	N/A	N/A	N/A	N/A
Caputo et al., Italy, 2000. [34]	54	Digital RadioVisioGraphy (RVG)	Neural network	N/A	N/A	N/A	N/A

**Table 2:** Classification results by both methods.

	Healthy					Unhealthy				
AI-based method				False Negative					False Positive	
			
	46		47	1		12		13	1	
	True Negative					True Positive				
			
Ground truth method	47					13				

## Data Availability

The data that support the findings of this study are available from the corresponding author upon reasonable request.
